# Short-Term Exposure to Fine Particulate Matter and Nitrogen Dioxide and Mortality in 4 Countries

**DOI:** 10.1001/jamanetworkopen.2023.54607

**Published:** 2024-03-01

**Authors:** Yiqun Ma, Federica Nobile, Anne Marb, Robert Dubrow, Massimo Stafoggia, Susanne Breitner, Patrick L. Kinney, Kai Chen

**Affiliations:** 1 Department of Environmental Health Sciences, Yale School of Public Health, New Haven, Connecticut; 2 Yale Center on Climate Change and Health, Yale School of Public Health, New Haven, Connecticut; 3 Department of Epidemiology, Lazio Region Health Service ASL Roma 1, Rome, Italy; 4 Chair of Epidemiology, Institute for Medical Information Processing, Biometry, and Epidemiology, Faculty of Medicine, Ludwig-Maximilians-Universität München, Munich, Germany; 5 Institute of Epidemiology, Helmholtz Zentrum München–German Research Center for Environmental Health, Neuherberg, Germany; 6 Department of Environmental Health, Boston University School of Public Health, Boston, Massachusetts

## Abstract

**Importance:**

The association between short-term exposure to air pollution and mortality has been widely documented worldwide; however, few studies have applied causal modeling approaches to account for unmeasured confounders that vary across time and space.

**Objective:**

To estimate the association between short-term changes in fine particulate matter (PM_2.5_) and nitrogen dioxide (NO_2_) concentrations and changes in daily all-cause mortality rates using a causal modeling approach.

**Design, Setting, and Participants:**

This cross-sectional study used air pollution and mortality data from Jiangsu, China; California; central-southern Italy; and Germany with interactive fixed-effects models to control for both measured and unmeasured spatiotemporal confounders. A total of 8 963 352 deaths in these 4 regions from January 1, 2015, to December 31, 2019, were included in the study. Data were analyzed from June 1, 2021, to October 30, 2023.

**Exposure:**

Day-to-day changes in county- or municipality-level mean PM_2.5_ and NO_2_ concentrations.

**Main Outcomes and Measures:**

Day-to-day changes in county- or municipality-level all-cause mortality rates.

**Results:**

Among the 8 963 352 deaths in the 4 study regions, a 10-μg/m^3^ increase in daily PM_2.5_ concentration was associated with an increase in daily all-cause deaths per 100 000 people of 0.01 (95% CI, 0.001-0.01) in Jiangsu, 0.03 (95% CI, 0.004-0.05) in California, 0.10 (95% CI, 0.07-0.14) in central-southern Italy, and 0.04 (95% CI, 0.02- 0.05) in Germany. The corresponding increases in mortality rates for a 10-μg/m^3^ increase in NO_2_ concentration were 0.04 (95% CI, 0.03-0.05) in Jiangsu, 0.03 (95% CI, 0.01-0.04) in California, 0.10 (95% CI, 0.05-0.15) in central-southern Italy, and 0.05 (95% CI, 0.04-0.06) in Germany. Significant effect modifications by age were observed in all regions, by sex in Germany (eg, 0.05 [95% CI, 0.03-0.06] for females in the single-pollutant model of PM_2.5_), and by urbanicity in Jiangsu (0.07 [95% CI, 0.04-0.10] for rural counties in the 2-pollutant model of NO_2_).

**Conclusions and Relevance:**

The findings of this cross-sectional study contribute to the growing body of evidence that increases in short-term exposures to PM_2.5_ and NO_2_ may be associated with increases in all-cause mortality rates. The interactive fixed-effects model, which controls for unmeasured spatial and temporal confounders, including unmeasured time-varying confounders in different spatial units, can be used to estimate associations between changes in short-term exposure to air pollution and changes in health outcomes.

## Key Points

**Question:**

What are the associations between short-term changes in fine particulate matter (PM_2.5_) and nitrogen dioxide (NO_2_) concentrations and changes in daily all-cause mortality rates?

**Findings:**

This cross-sectional study of more than 8.9 million deaths found that a 10-μg/m^3^ increase in daily PM_2.5_ concentrations was associated with increases in daily all-cause deaths per 100 000 people of 0.01 in Jiangsu, China, 0.03 in California, 0.10 in central-southern Italy, and 0.04 in Germany; corresponding increases in mortality rates for the same increase in NO_2_ concentrations were 0.04, 0.03, 0.10, and 0.05, respectively.

**Meaning:**

The findings of this study add to the growing evidence that increases in short-term exposures to PM_2.5_ and NO_2_ may be associated with increases in mortality rates.

## Teaser

This cross-sectional study estimates the association between changes in daily concentrations of fine particulate matter (PM_2.5_) and nitrogen dioxide (NO_2_) and changes in daily mortality rates in China, the United States, Italy, and Germany using an interactive fixed-effects approach.

## Introduction

The association between short-term exposure to air pollution and mortality has been widely documented worldwide.^1,2,3,4^ Most previous epidemiological studies have investigated the effects of short-term exposure to air pollution on mortality using time-series analyses.^1,2,3^ By contrasting each spatial unit to itself over time, time-series studies avoid the confounding effects of spatial and time-invariant variables by design; however, results from time-series analyses may be biased due to unmeasured temporal confounding.^5,6,7^ Alternative causal modeling approaches that account for unmeasured time-varying confounders in different spatial units offer an attractive alternative for studying the association between short-term air pollution exposure and mortality.

Fixed-effects models, particularly 2-way fixed-effects (TWFE) models, have been widely applied for causal inference in econometrics and other social sciences.^8^ In the field of air pollution epidemiology, TWFE models have been recently applied, first in studies of long-term air pollution exposure^9,10,11^ and most recently to estimate the effects of short-term fine particulate matter (PM_2.5_) exposure on hospitalizations.^7^ By introducing indicators for each spatial unit and each time unit, TWFE models can control for all spatial confounders that vary across space but not across the study timescales (eg, geographical and socioeconomic factors) and all temporal confounders that vary by time but not space (eg, day of week and seasonality).^9,12^ However, this approach assumes no unmeasured confounders that display different temporal variations across spatial units (time-varying spatial unit effects), an assumption that is often violated.^13^ To relax this assumption of the TWFE model, the more flexible interactive fixed-effects (IFE) model was developed. By decomposing the unmeasured time-varying spatial unit–specific confounders into heterogeneous effects of common trends, the IFE model can potentially control for such confounders in the regression.^13,14,15^ To the best of our knowledge, this study is the first application of the IFE approach to model the causal effects of short-term exposure to air pollution on mortality.

Substantial heterogeneity in the association between air pollution concentrations and daily mortality exists across countries and regions,^1,2,3^ especially between developed and developing countries and between regions with low and high levels of pollution.^1,3^ A multicountry approach has the potential to comprehensively describe the effects of air pollution in regions with different air pollution levels and socioeconomic statuses. This study focused on 4 study regions: Jiangsu Province, China (hereinafter Jiangsu); California; central-southern Italy; and Germany. We specifically examined PM_2.5_ and nitrogen dioxide (NO_2_) as 2 major traffic- and/or health-related air pollutants. Using data from these 4 regions from January 1, 2015, to December 31, 2019, we estimated the association between changes in daily PM_2.5_ and NO_2_ concentrations and changes in daily mortality rates using an IFE approach. Potential effect modifications by sex, age, and urbanicity were further examined.

## Methods

This cross-sectional study used anonymized daily county- or municipality-level mortality records and was approved by the Yale Institutional Review Board. The need for informed consent was waived owing to the use of publicly available deidentfied data. This study followed the Strengthening the Reporting of Observational Studies in Epidemiology (STROBE) reporting guideline.

### Mortality Rate

We collected daily all-cause mortality data from 2015 to 2019 by sex and age in each spatial unit (county in Jiangsu, California, and Germany and municipality in central-southern Italy) of each region (eAppendix 1 in Supplement 1). Based on the population size of each spatial unit (eAppendix 1 in Supplement 1), we calculated daily spatial unit–level all-cause mortality rates for the whole population and population subgroups by sex (male and female) and age (0-74 and ≥75 years). In central-southern Italy, to have a sufficient number of deaths in each included municipality, we included in the analysis only the 669 municipalities with a population of 10 000 or greater of a total of 3250 municipalities. This population cutoff was only applied to central-southern Italy because all counties in the other 3 regions satisfied this population criterion.

### Air Pollution and Meteorological Variables

Daily concentrations of PM_2.5_ and NO_2_ at 1 × 1-km^2^ resolution from 2015 to 2019 were estimated by spatiotemporal models for Jiangsu,^16,17^ central-southern Italy,^18,19^ and Germany^20,21^ (eAppendix 2 in Supplement 1). In brief, the models incorporated variables from satellite observations, chemical transport model simulations, land use and/or land cover characteristics, and meteorological data as predictive factors and applied advanced machine learning algorithms to model daily air pollution concentrations. The modeled air pollution data were aggregated to daily county- or municipality-level concentrations using the geographic mean and matched to the mortality data.

To the best of our knowledge, there was no available high-resolution daily spatiotemporal model for PM_2.5_ and NO_2_ covering 2015 to 2019 for California at the time of our study, so we relied on the records of air quality monitoring sites managed by the US Environmental Protection Agency Air Quality System.^22^ The 32 California counties with monitoring sites for both PM_2.5_ and NO_2_ were included in the analysis (of 58 counties), including data from 108 sites for PM_2.5_ and 115 sites for NO_2_. For counties in California with multiple monitoring sites, we calculated county-level mean air pollution concentrations and used these in our analyses.

Hourly air temperature data at 0.1° × 0.1° resolution were extracted from the ERA5-Land reanalysis dataset^23^ for all 4 regions. Like the air pollution data, we calculated the mean air temperature data for each day in each spatial unit. We also calculated the daily dew point temperature and relative humidity in each region (eAppendix 3 in Supplement 1).

### Statistical Analysis

Data were analyzed from June 1, 2021, to October 30, 2023. The IFE model assumes that the unmeasured spatial unit effects that change over time have a factor structure and that all regressors and factors are stationary. Additionally, the IFE model allows a weak serial and cross-sectional correlation.^14^ In our study, the IFE model was expressed as Δmortality rate*_i,t_* = μ + α*_i_* + βΔair pollution*_i,t_* + *ns*(Δtemperature*_i,t_*, *df* = 5) + *v_i,t_* + ε*_i,t_*, where ν*_i_*_,_*_t_* = Σ*^d^_l_*_ = 1_λ*_i_*_,_*_l_f_l_*_,_*_t_*, the outcome variable Δmortality rate*_i,t_* is the change in all-cause mortality rate from day *t* – 1 to day *t* in spatial unit *i*, and Δair pollution*_i,t_* is the change in mean concentration of PM_2.5_ or NO_2_ from day *t* – 1 to day *t* in spatial unit *i*. The day-to-day change in air temperature on the same lag day as the air pollution variable (Δtemperature*_i,t_*) was included as a flexible natural cubic spline with 5 *df*. First-order differences of the observed time series were taken to remove the long-term and seasonal trend of mortality rate, air pollution, and meteorological factors and to meet the stationarity and normality assumptions of the model. The α*_i_* refers to time-invariant spatial unit effects, which can help control for the effects of spatial confounders that do not vary over time. The μ is the intercept; ε*_i,t_* is the error term; and *v_i,t_* is the unmeasured time-varying spatial unit effect, which is decomposed into *d* common time-varying factors *f_l,t_*, with corresponding unobserved spatial unit–level loading parameters λ*_i,l_.*^24,25^ The number of factors (*d*) was selected following the criteria proposed by Bai and Ng^24^ (eAppendix 4 in Supplement 1). The coefficient of changes in air pollution (β) represents the expected change in the daily mortality rate for each unit change in the daily air pollution level. Heteroskedasticity and autocorrelation-consistent standard errors (SEs) were calculated to account for potential heteroskedasticity and autocorrelation in the error terms. The sample R code for our main IFE model can be found in eAppendix 5 in Supplement 1.

To account for the potential confounding effects from the other air pollutant, we also used 2-pollutant models in which both PM_2.5_ and NO_2_ were included. We explored the lag pattern in the associations of PM_2.5_ and NO_2_ changes with changes in daily mortality rate on the current day and the previous 2 days using both single lag days (lag0 to lag2) and cumulative lag days (lag01 to lag02), which was commonly used in previous studies.^1,2,3^ The IFE analyses were performed with R software, version 4.1.3 (R Project for Statistical Computing) using the package phtt.^25^

We conducted subgroup analyses to estimate potential differences in the effects of air pollution according to sex (male and female) and age (0-74 years and ≥75 years). In addition, we performed stratified analyses to examine the potential effect modification by urbanicity (urban and rural). The classification criteria of urban and rural areas in each study region are described in eAppendix 6 in Supplement 1. We tested the statistical difference in effect estimates between groups by calculating the *z* score as (Q̂_1_ − Q̂_2_)/√( SÊ_1_)^2^ + (SÊ_2_)^2^, where Q̂_1_ and Q̂_2_ are the estimates and SÊ_1_ and SÊ_2_ are their respective standard errors.^26^ Statistical significance was evaluated at *P* < .05 (2-sided test).

To evaluate the model specification, we performed a randomization test in which we randomized the PM_2.5_ or NO_2_ concentration 2000 times across all 1825 days in each spatial unit. Such a randomization test is widely applied to detect temporal dependence in panel models due to model misspecification.^27^ In addition, we performed several sensitivity analyses to test the robustness of our results: (1) we additionally adjusted for relative humidity or dew point temperature in the model; (2) we applied a traditional fixed-effects model, which does not consider unmeasured time-varying spatial unit effects; and (3) we used 4 *df* or 6 *df* instead of 5 *df* in the natural cubic spline of air temperature. Furthermore, we compared the results from the IFE model with those from a traditional 2-stage time-series model (eAppendix 7 in Supplement 1).^2,3^

## Results

### Description of Mortality Rate and Air Pollution Exposure

**Figure 1.**
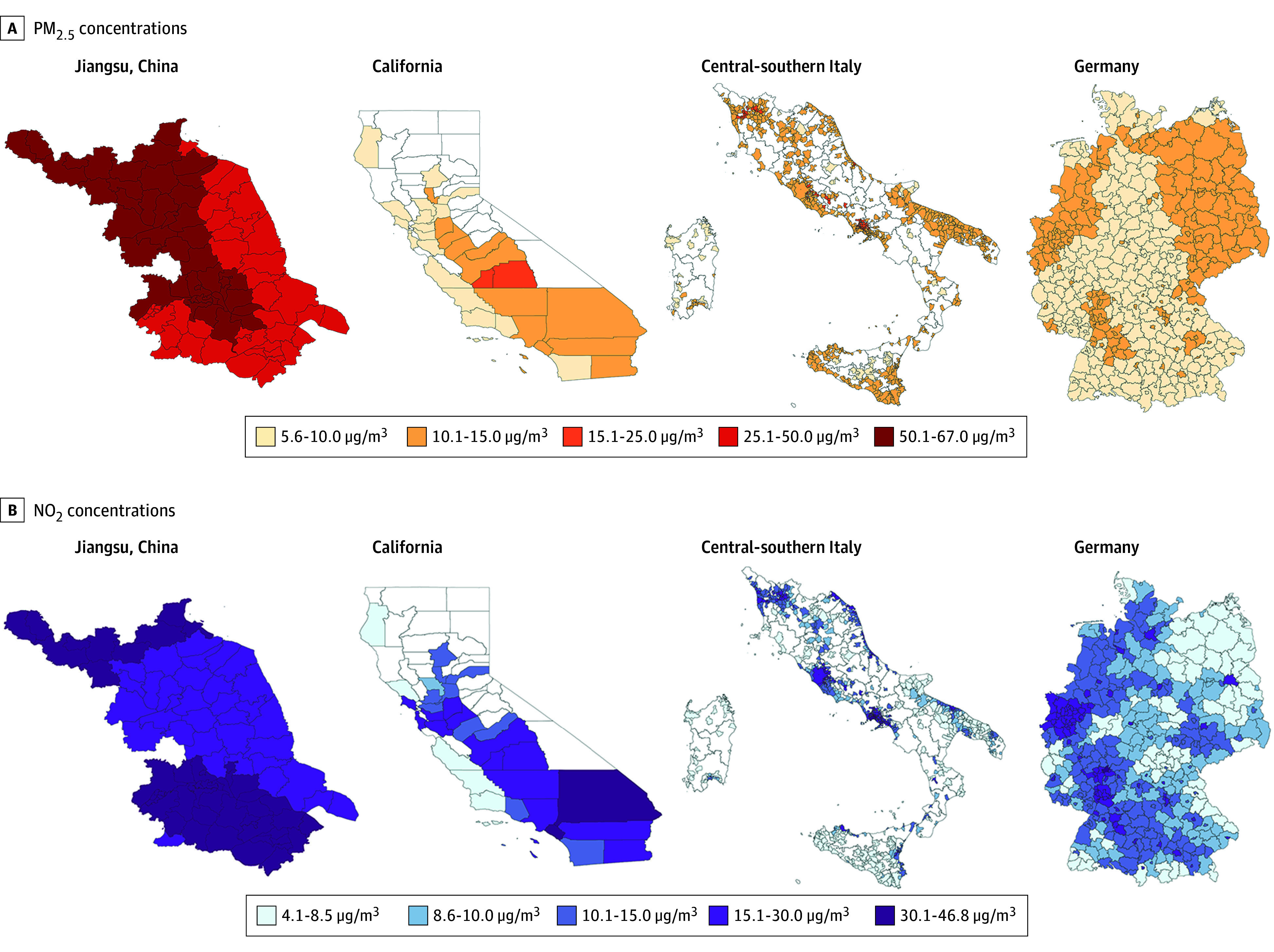
Daily Mean Fine Particulate Matter (PM_2.5_) and Nitrogen Dioxide (NO_2_) Concentrations in Each Spatial Unit in All Study Regions The blank areas in California represent counties that lack air quality monitoring stations for both PM_2.5_ and NO_2_; the blank areas in central-southern Italy represent municipalities with a population of less than 10 000 individuals.

This study covered a total of 8 963 352 deaths, including 2 633 920 in Jiangsu (45.0% female and 55.0% male; 60.0% aged ≥75 years), 1 237 862 in California (48.3% female and 51.7% male; 58.7% aged ≥75 years), 1 227 482 in central-southern Italy (51.8% female and 48.2% male; 73.5% aged ≥75 years), and 3 864 088 in Germany (52.1% female and 47.9% male; 72.5% aged ≥75 years) from 2015 to 2019. The descriptive statistics of daily mortality rate, PM_2.5_ and NO_2_ concentrations, and air temperature in each region are presented in Table 1. No data were missing in this study. The mean (SD) spatial unit–level PM_2.5_ and NO_2_ concentrations were the highest in Jiangsu (PM_2.5_, 50.8 [27.8] μg/m^3^; NO_2_, 32.2 [1.28] μg/m^3^). The mean (SD) PM_2.5_ concentration was the lowest in California (9.9 [9.2] μg/m^3^), and the mean (SD) NO_2_ concentration was the lowest in Germany (12.2 [7.8] μg/m^3^) (Table 1). The spatial distribution of PM_2.5_ and NO_2_ in the 4 study regions is displayed in Figure 1.

**Table 1.** Descriptive Statistics of Daily County- or Municipality-Level Mortality Rate and Environmental Data, 2015 to 2019

Study region	Mean (SD)	Median (IQR) [range]
**Jiangsu, China** ^a^		
All-cause mortality rate^b^		
All-group	1.9 (0.7)	1.8 (0.9) [0.0 to 11.2]
Male	2.0 (1.0)	1.9 (1.2) [0.0 to 11.4]
Female	1.7 (0.9)	1.6 (1.1) [0.0 to 13.5]
Aged 0-74 y	0.8 (0.4)	0.7 (0.5) [0.0 to 5.9]
Aged ≥75	26.7 (13.5)	24.6 (16.1) [0.0 to 210.7]
Environmental factors		
PM_2.5_ concentration, μg/m^3^	50.8 (27.8)	44.0 (31.8) [6.0 to 254.4]
NO_2_ concentration, μg/m^3^	32.2 (12.8)	29.8 (15.1) [6.6 to 122.5]
Air temperature, °C	16.1 (9.1)	17.1 (15.7) [−10.0 to 34.8]
**California** ^c^		
All-cause mortality rate^b^		
All-group	2.0 (0.8)	1.9 (0.8) [0.0 to 9.5]
Male	2.0 (1.1)	1.9 (1.1) [0.0 to 14.7]
Female	1.9 (1.1)	1.8 (1.0) [0.0 to 10.4]
Aged 0-74 y	0.9 (0.6)	0.8 (0.6) [0.0 to 6.7]
Aged ≥75 y	19.8 (10.0)	18.7 (10.4) [0.0 to 108.5]
Environmental factors		
PM_2.5_ concentration, μg/m^3^	9.9 (9.2)	8.0 (6.4) [0.0 to 411.7]
NO_2_ concentration, μg/m^3^	15.7 (11.4)	12.8 (13.6) [0.0 to 108.2]
Air temperature, °C	16.3 (7.0)	15.7 (10.1) [−5.0 to 40.0]
**Central-southern Italy** ^d^		
All-cause mortality rate^b^		
All-group	2.7 (3.9)	0.0 (4.7) [0.0 to 69.9]
Male	2.7 (5.5)	0.0 (3.4) [0.0 to 106.0]
Female	2.7 (5.3)	0.0 (3.5) [0.0 to 79.0]
Aged 0-74 y	0.8 (2.2)	0.0 (0.0) [0.0 to 43.6]
Aged ≥75 y	19.2 (32.7)	0.0 (31.6) [0.0 to 645.7]
Environmental factors		
PM_2.5_ concentration, μg/m^3^	12.6 (6.0)	11.3 (5.7) [1.3 to 99.3]
NO_2_ concentration, μg/m^3^	12.4 (7.3)	10.1 (7.6) [1.8 to 81.2]
Air temperature, °C	15.9 (6.8)	15.5 (10.6) [−12.7 to 35.3]
**Germany** ^e^		
All-cause mortality rate^b^		
All-group	2.6 (1.6)	2.4 (1.9) [NA]
Male	2.5 (2.1)	2.2 (2.5) [NA]
Female	2.7 (2.2)	2.4 (2.5) [NA]
Aged 0-74 y	0.8 (0.9)	0.7 (1.2) [NA]
Aged ≥75 y	16.3 (10.9)	15.3 (13.3) [NA]
Environmental factors		
PM_2.5_ concentration, μg/m^3^	10.0 (7.1)	8.2 (6.8) [0.5 to 141.7]
NO_2_ concentration, μg/m^3^	12.2 (7.8)	10.1 (8.8) [0.6 to 83.9]
Air temperature, °C	10.2 (7.4)	10.0 (11.9) [−17.9 to 31.8]

Abbreviations: NA, not available; NO_2_, nitrogen dioxide; PM_2.5_, fine particulate matter.

^a^ Includes 82 counties with a total population of 77 955 026 persons.

^b^ Reported per 100 000 population.

^c^ Includes the 32 out of 58 counties with Environmental Protection Agency air quality monitoring stations, with a total population of 37 290 255.

^d^ Includes 669 of 3250 municipalities with a population of 10 000 or greater, with a total population of 24 545 330.

^e^ Includes 401 counties with a total population of 82 735 005. Minimum and maximum values of mortality rates in Germany were not allowed to be released according to the information protection policies of the German Statistical Offices.

### Association Between Short-Term Air Pollution Exposure and Mortality Rate

**Figure 2.**
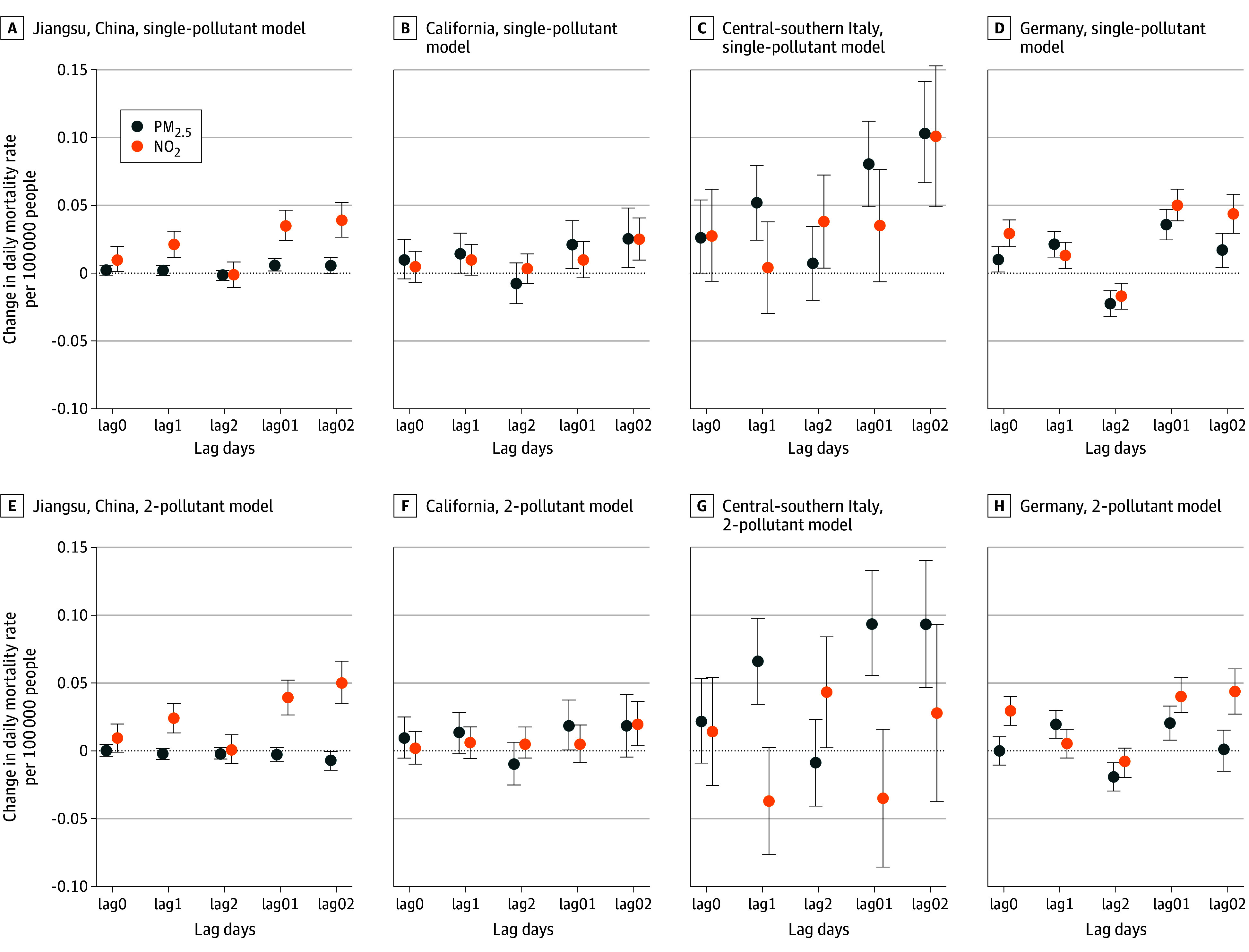
Estimated Change in Daily Mortality Rate Associated With a 10-μg/m^3^ Increase in Fine Particulate Matter (PM_2.5_) or Nitrogen Dioxide (NO_2_) Concentration Single- and 2-pollutant models on different lag days in each study region are shown. The error bars represent the 95% CIs. Lag0 to lag2 represent single lag days; lag01 to lag02, cumulative lag days.

Figure 2 shows the estimated associations between changes in daily PM_2.5_ and NO_2_ concentrations and changes in daily mortality rate (per 100 000 people) from both single- and 2-pollutant models in each region. In single-pollutant models, increases in PM_2.5_ and NO_2_ concentrations were associated with increases in mortality rates in all 4 study regions. We define the main lag for each pollutant in each region as the lag with the greatest effect size from the single-pollutant model; the estimated coefficients for all lags are presented in eTable 1 in Supplement 1. In the single-pollutant models for PM_2.5_ in Jiangsu and Germany, the greatest effect estimates were observed for lag01; a 10-μg/m^3^ increase in 2-day mean concentration of PM_2.5_ was associated with increases in daily all-cause deaths per 100 000 people of 0.01 (95% CI, 0.001-0.01) in Jiangsu and 0.04 (95% CI, 0.02-0.05) in Germany. In California and central-southern Italy, the highest estimate for PM_2.5_ in the single-pollutant model was found for lag02; a 10-μg/m^3^ increase in 3-day mean PM_2.5_ concentrations was associated with increases in daily all-cause deaths per 100 000 people of 0.03 (95% CI, 0.004-0.05) in California and 0.10 (95% CI, 0.07-0.14) in central-southern Italy. In the single-pollutant models, the largest effect estimates for a 10-μg/m^3^ increase in NO_2_ concentration were 0.04 (95% CI, 0.03-0.05) deaths per 100 000 people in Jiangsu (lag02), 0.03 (95% CI, 0.01-0.04) deaths per 100 000 people in California (lag02), 0.10 (95% CI, 0.05-0.15) deaths per 100 000 people in central-southern Italy (lag02), and 0.05 (95% CI, 0.04-0.06) deaths per 100 000 people in Germany (lag01). The results from 2-pollutant models were generally consistent with those from single-pollutant models, though the estimated coefficients were relatively smaller (Figure 2 and eTable 1 in Supplement 1).

### Effect Modification by Sex, Age, and Urbanicity

We used the lags with the largest effect sizes in each region to examine potential effect modification. In subgroup analyses by sex, we found a significantly larger effect size in the association between changes in daily PM_2.5_ concentration and changes in daily mortality rate among females compared with males in Germany in both single-pollutant (0.05 [95% CI, 0.03-0.06] deaths per 100 000 people; *P* = .03) and 2-pollutant (0.04 [95% CI, 0.02-0.05] deaths per 100 000 people; *P* = .01) models (Table 2). No statistically significant differences between sexes were observed for PM_2.5_ in other regions or for NO_2_.

**Table 2.** Estimated Changes in Daily Mortality Rate Associated With a 10-μg/m^3^ Increase in PM_2.5_ and NO_2_ Concentrations by Sex and Age^a^

Subgroup	Single-pollutant model				Two-pollutant model				
	PM_2.5_		NO_2_		PM_2.5_		NO_2_		
	Estimated changes in daily mortality rate (95% CI)^b^	*P* value^c^	Estimated changes in daily mortality rate (95% CI)^b^	*P* value^c^	Estimated changes in daily mortality rate (95% CI)^b^	*P* value^c^	Estimated changes in daily mortality rate (95% CI)^b^		*P* value^c^
**Jiangsu, China**									
Sex									
Male	0.01 (0.003 to 0.01)	.51	0.04 (0.03 to 0.05)	.83	−0.0006 (−0.01 to 0.004)	.31		0.05 (0.03 to 0.06)	.39
Female	0.01 (0.001 to 0.01)		0.04 (0.03 to 0.05)		−0.004 (−0.01 to 0.0004)			0.05 (0.04 to 0.07)	
Age, y									
0-74	0.008 (−0.0009 to 0.002)	.10	0.01 (0.01 to 0.02)	<.001	−0.002 (−0.004 to 0.00006)	.78		0.02 (0.01 to 0.02)	<.001
≥75	0.06 (−0.01 to 0.13)		0.46 (0.23 to 0.69)		−0.01 (−0.10 to 0.07)			0.61 (0.30 to 0.91)	
**California**									
Sex									
Male	0.03 (−0.003 to 0.06)	.79	0.02 (−0.01 to 0.04)	.25	0.02 (−0.01 to 0.05)	.94		0.01 (−0.01 to 0.04)	.27
Female	0.03 (0.003 to 0.06)		0.04 (0.01 to 0.06)		0.02 (−0.01 to 0.05)			0.03 (0.01 to 0.05)	
Age, y									
0-74	0.02 (0.01 to 0.03)	.20	0.004 (−0.004 to 0.01)	<.001	0.02 (0.01 to 0.03)	.78		−0.0009 (−0.01 to 0.01)	<.001
≥75	0.20 (−0.08 to 0.47)		0.40 (0.19 to 0.61)		0.06 (−0.22 to 0.34)			0.39 (0.17 to 0.60)	
**Central-southern Italy**									
Sex									
Male	0.09 (0.03 to 0.14)	.37	0.08 (0.01 to 0.16)	.50	0.08 (0.01 to 0.14)	.55		0.02 (−0.07 to 0.11)	.83
Female	0.12 (0.07 to 0.17)		0.12 (0.05 to 0.19)		0.11 (0.04 to 0.17)			0.04 (−0.06 to 0.13)	
Age, y									
0-74	0.01 (−0.01 to 0.04)	<.001	0.03 (−0.0006 to 0.06)	<.001	0.002 (−0.02 to 0.03)	<.001		0.03 (−0.01 to 0.07)	.45
≥75	1.00 (0.65 to 1.35)		0.98 (0.45 to 1.51)		0.90 (0.46 to 1.33)			0.28 (−0.37 to 0.93)	
**Germany**									
Sex									
Male	0.02 (0.01 to 0.04)	.03	0.05 (0.04 to 0.07)	.79	0.004 (−0.01 to 0.02)	.01		0.05 (0.03 to 0.07)	.19
Female	0.05 (0.03 to 0.06)		0.05 (0.03 to 0.07)		0.04 (0.02 to 0.05)			0.03 (0.01 to 0.05)	
Age, y									
0-74	0.01 (0.01 to 0.02)	<.001	0.02 (0.01 to 0.02)	<.001	0.01 (0.001 to 0.01)	.03		0.01 (0.01 to 0.02)	<.001
≥75	0.21 (0.13 to 0.29)		0.31 (0.22 to 0.40)		0.11 (0.02 to 0.20)			0.26 (0.17 to 0.36)	

Abbreviations: NO_2_, nitrogen dioxide; PM_2.5_, fine particulate matter.

^a^ In subgroup analyses, we used the main lag day (lag01 to lag02 represent cumulative lag days) for each pollutant in each region (Jiangsu: PM_2.5_ lag01, NO_2_ lag02; California: PM_2.5_ lag02, NO_2_ lag02; central-southern Italy: PM_2.5_ lag02, NO_2_ lag02; and Germany: PM_2.5_ lag01, NO_2_ lag01).

^b^ Calculated per 100 000 population.

^c^ We tested the statistical differences in effect estimates between males and females and between those aged 0 to 74 years and those 75 years or older based on the *z* score calculated using the coefficients and standard errors for different groups.

In subgroup analyses by age, changes in daily PM_2.5_ concentration and changes in daily mortality rate were found to be significantly greater among people 75 years and older than among those aged 0 to 74 years in central-southern Italy for the single-pollutant model (1.00 [95% CI, 0.65-1.35] deaths per 100 000 people; *P* < .001) and the 2-pollutant model (0.90 [95% CI, 0.46-1.33 deaths per 100 000 people; *P* < .001) and in Germany for the single-pollutant model (0.21 [95% CI, 0.13-0.29] deaths per 100 000 people; *P* < .001) and the 2-pollutant model (0.11 [95% CI, 0.02-0.20] deaths per 100 000 people; *P* = .03). Consistently, for NO_2_, the association was greater among people 75 years and older compared with those aged 0 to 74 years in all 4 regions in single-pollutant models (0.46 [95% CI, 0.23-0.69] deaths per 100 000 people in Jiangsu; 0.40 [95% CI, 0.19-0.61] deaths per 100 000 people in California; 0.98 [95% CI, 0.45-1.51] deaths per 100 000 people in central-southern Italy; and 0.31 [95% CI, 0.22-0.40] deaths per 100 000 people in Germany) (*P* < .001 for all) and in Jiangsu (0.61 [95% CI, 0.30-0.91] deaths per 100 000 people), California (0.39 [95% CI, 0.17-0.60] deaths per 100 000 people), and Germany (0.26 [95% CI, 0.17-0.36] deaths per 100 000 people) in 2-pollutant models (*P* < .001 for all) (Table 2).

**Figure 3.**
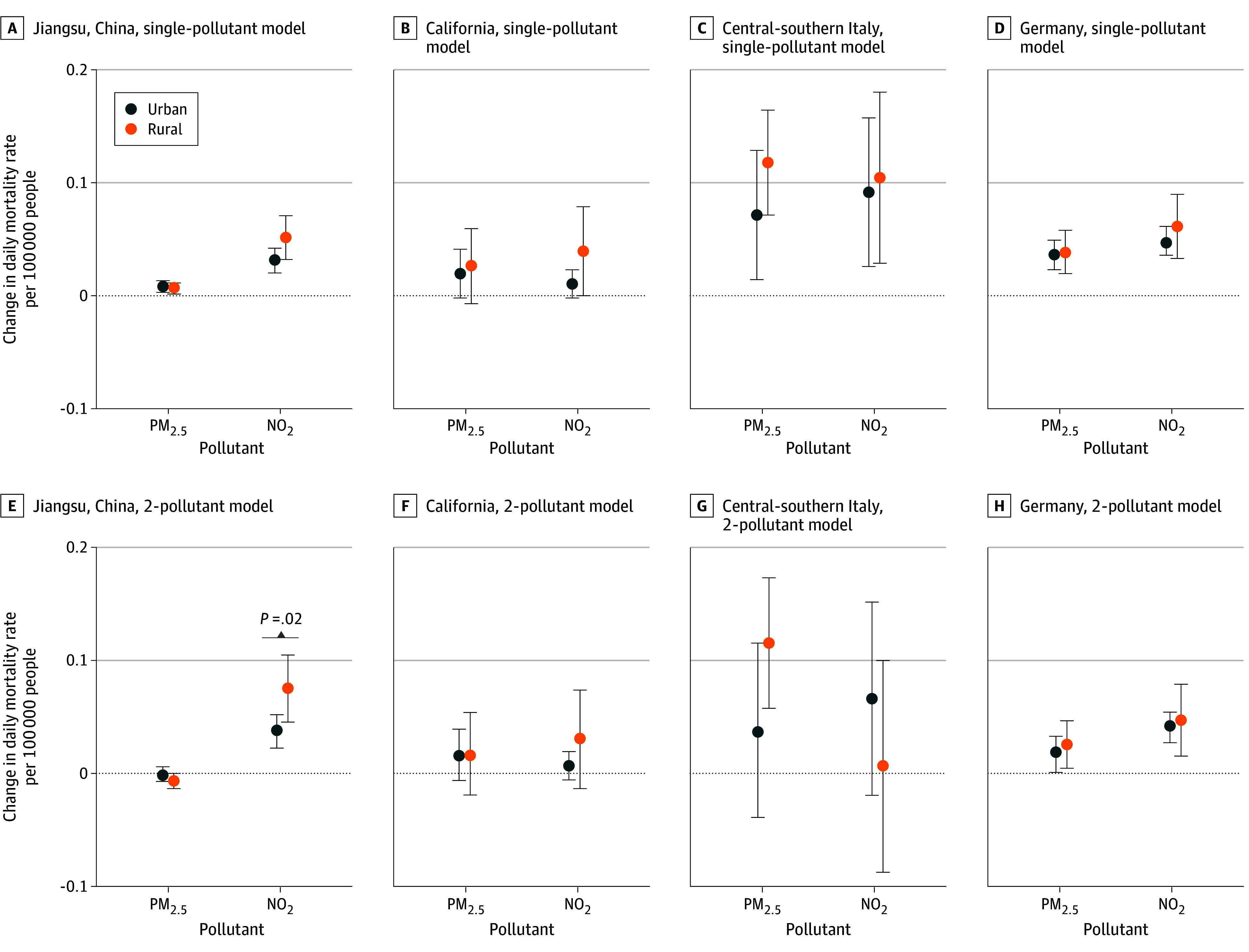
Estimated Change in Daily Mortality Rate Associated With a 10-μg/m^3^ Increase in Fine Particulate Matter (PM_2.5_) or Nitrogen Dioxide (NO_2_) Concentration in Urban and Rural Areas Single- and 2-pollutant models in urban and rural areas in each study region are shown. The error bars represent the 95% CIs. The lag with the strongest pollutant-mortality association for each pollutant in each region was used in the stratified analysis (Jiangsu: PM_2.5_ lag01, NO_2_ lag02; California: PM_2.5_ lag02, NO_2_ lag02; central-southern Italy: PM_2.5_ lag02, NO_2_ lag02; Germany: PM_2.5_ lag01, NO_2_ lag01). We tested the statistical differences in effect estimates between urban and rural areas by calculating *P* values based on the *z* score derived from the coefficients and SEs for different subgroups. *P* values were noted only for statistically significant between-group differences.

In stratified analyses by urbanicity, we found no statistically significant differences in the association between changes in daily PM_2.5_ or NO_2_ concentration and changes in daily mortality rate between urban and rural areas except for NO_2_ in Jiangsu, where the difference was significant in rural compared with urban areas in the 2-pollutant model (0.07 [95% CI, 0.04-0.10] deaths per 100 000 people; *P* = .02) (Figure 3 and eTable 2 in Supplement 1).

### Randomization Test, Sensitivity Analysis, and Comparative Analysis

As shown in eFigure 1 in Supplement 1, the distributions of the model coefficients when the changes in PM_2.5_ or NO_2_ concentrations were randomly assigned 2000 times across days were centered at approximately 0 in all 4 regions, and the coefficient estimates from our main model fell substantially outside these distributions in all study regions. This indicates that the estimated associations between air pollution and mortality in our study were unlikely driven by temporal dependence due to a misspecification of the model.

Sensitivity analyses showed that our results remained robust when additionally adjusting for dew point temperature and relative humidity using traditional fixed-effects models and alternative degrees of freedom for air temperature (eTable 3 in Supplement 1). The estimated unmeasured time-varying spatial unit effects in the main IFE model for the main lag in Jiangsu and California are visualized in eFigure 2 in Supplement 1. We observed 1 unmeasured factor that displayed different temporal variations across spatial units in Jiangsu and 5 in California. However, these factors may not be directly interpretable because they are, at best, linear transformations of the true factors. No such factors were detected by the IFE model in central-southern Italy and Germany.

The results from the traditional 2-stage time-series analysis are shown in eFigure 3 in Supplement 1. While the estimates from the IFE model and those from the time-series analysis should not be directly compared because of their different interpretations, the positive estimates from each analysis reinforce the findings from the IFE model. Both suggested that short-term exposure to PM_2.5_ or NO_2_ was associated with an increase in all-cause mortality.

## Discussion

Using the IFE model, a causal modeling approach, we found that short-term increases in PM_2.5_ and NO_2_ concentrations were associated with increases in daily mortality rate in Jiangsu, California, central-southern Italy, and Germany. The IFE model has the advantage of controlling for unobserved confounding factors that vary over time within each spatial unit. This study showcases the applicability of the IFE approach across diverse regions, including China, the US, and Europe, encompassing varying levels of air pollution concentrations and socioeconomic statuses.

Our estimated associations between short-term changes in air pollution and changes in daily mortality rates in 4 countries are generally consistent with findings from previous studies using time-series analysis.^1,2,3^ For example, in the multicountry and multicity studies,^2,3^ a 10-μg/m^3^ increase in PM_2.5_ concentration on lag01 or in NO_2_ concentration on lag1 was found to be associated with a 0.68% or 0.46% increase in daily all-cause mortality, respectively. In a study based on 272 cities in China, a 10-μg/m^3^ increase in the 2-day mean NO_2_ concentration was associated with an increase of 0.9% in daily total nonaccidental mortality.^1^ However, the estimates of our study cannot be directly compared with those from time-series analyses due to different interpretations of the model coefficients. The coefficient in our model estimates the change in the daily mortality rate for each 10-μg/m^3^ change in the daily air pollution level; the coefficient does not directly provide information about the association between the absolute level of air pollution and the absolute level of mortality. Nevertheless, the consistency between the results from traditional time-series analyses and the novel IFE approach provides robust support for an association between short-term exposure to PM_2.5_ and NO_2_ and mortality.

In addition, the results from our main IFE models were similar to those from traditional fixed-effects models, which do not take into account unmeasured time-varying effects at the spatial unit level (eTable 3 in Supplement 1). This suggests that even though we observed such unmeasured spatial unit–specific temporal confounding (as seen in the model for the main lags in Jiangsu and California) (eFigure 2 in Supplement 1), its role in the estimated air pollution–mortality association was minimal. This observation further bolsters our confidence in traditional time-series analysis. Despite its inherent limitations in adjusting for unmeasured temporal confounding, the results it produces remain valid.

Although short-term exposure to NO_2_ has been linked to mortality and morbidity outcomes by numerous epidemiological studies,^28,29^ questions remain about the causal nature of this association.^30,31,32^ As stated in the Integrated Science Assessment by the US Environmental Protection Agency, current evidence for short-term NO_2_ exposure is “suggestive of, but not sufficient to infer, a causal relationship.”^30^ Because NO_2_ and other combustion-derived air pollutants such as PM_2.5_ can be coemitted from traffic and other sources,^33,34^ it has been speculated that NO_2_ only serves as a surrogate for other traffic-related air pollutants.^34,35,36^ This uncertainty not only calls into question the weight of epidemiological evidence on the health effects of NO_2_ but also complicates policy making for air quality regulations.^34^ In our study, the NO_2_ association remained after adjustment for PM_2.5_ in the 2-pollutant model in all 4 study regions, suggesting that NO_2_ may have its own independent effect on mortality that is not explained by PM_2.5_ levels.

We found substantial effect modification by age in all study regions. The effect modification of associations between short-term changes in air pollution and changes in daily mortality rate was significantly larger among people 75 years and older than among those aged 0 to 74 years. This association among older people reflects both the high mortality rate (Table 1) and the high susceptibility in the older population as found in previous studies.^37^

This study encompasses regions from 4 distinct countries spanning different air pollution levels and diverse socioeconomic backgrounds. Substantial heterogeneity in the associations between short-term changes in air pollution and changes in daily mortality rate was observed across study regions. These heterogeneous results could be explained by different chemical compositions of particulate matter, population characteristics (such as age structure) affecting susceptibility, regional climate, socioeconomic factors, and other factors.^38^ In addition, in central-southern Italy, the spatial unit was the municipality, which was much smaller than the counties used in other study regions. This resulted in fewer daily mortality counts in each spatial unit and, consequently, greater uncertainties in central-southern Italy. The difference in spatial scale also introduced different degrees of exposure measurement error; using larger spatial units such as counties might dilute the actual effects.

### Limitations

This study has several limitations. First, using county- or municipality-level air pollution and mortality data, we were unable to capture variations within individual spatial units, which may be particularly pronounced in larger counties or municipalities. Second, the air pollution exposures in Jiangsu, central-southern Italy, and Germany were modeled and therefore subject to uncertainty. In California, our reliance on records from air quality monitoring stations did not yield full spatial coverage. Third, although the IFE model relaxed the assumptions of the traditional TWFE model, it has its own assumptions, and violation of these assumptions might bias the results. In addition, we assumed a linear association between air pollution and mortality, but this assumption may oversimplify the actual relationship. Studies with finer spatial resolution, wider spatial coverage, and more accurate exposure assessment are needed to further investigate the potentially nonlinear causal relationship between short-term air pollution exposure and health outcomes.

## Conclusions

In this cross-sectional study, after controlling for both measured and unmeasured spatiotemporal confounders, we found that increases in short-term PM_2.5_ and NO_2_ concentrations were associated with increases in all-cause mortality rates. This study contributed to the growing body of evidence on the potentially detrimental health effects of short-term PM_2.5_ and NO_2_ exposure. The IFE model can be used to estimate associations between changes in short-term exposure to air pollution and changes in daily health outcomes.

## Notes

Supplement 1. **eAppendix 1. **Sources of Mortality and Population Data in Each Region **eAppendix 2. **Air Pollution Spatiotemporal Models for Jiangsu, China, Central-Southern Italy, and Germany **eAppendix 3. **Relative Humidity Calculation in Each Region **eAppendix 4. **Selection of the Number of Common Time-Varying Factors in the Unmeasured Time-Varying Spatial Unit Effect in the Interactive Fixed-Effects Model **eAppendix 5. **Sample R Code for the Main Interactive Fixed-Effects Model **eAppendix 6. **Classification of Urban and Rural Areas in Each Study Region **eAppendix 7. **Traditional 2-Stage Time-Series Analysis **eFigure 1. **Randomization Test for the Main Lag in Each Study Region **eFigure 2**. Plot of Estimated Common Time-Varying Factors and Unmeasured Time-Varying Spatial Unit Effects in the Main Single-Pollutant Models for PM_2.5_ or NO_2 _for Jiangsu, China, and California **eFigure 3**. Association Between Air Pollution and Mortality Estimated From Interactive Fixed-Effects Model and Traditional 2-Stage Time-Series Analysis **eTable 1. **Estimated Change in Daily Mortality Rate (per 100 000 People) Associated With a 10-µg/m^3^ Increase in PM_2.5_ or NO_2_ Concentration (95% CI) by Lag **eTable 2. **Estimated Change in Daily Mortality Rate (per 100 000 People) Associated With a 10-µg/m^3^ Increase in PM_2.5_ or NO_2_ Concentration by Urbanicity (95% CI) **eTable 3. **Estimated Changes in Daily Mortality Rate (per 100 000 People) Associated With a 10-µg/m^3^ Increase in PM_2.5_ and NO_2_ Concentrations in Sensitivity Analyses
eReferences.

Supplement 2.
Data Sharing Statement
