# Supplementary material for: Short-Term Exposure to Fine Particulate Matter and Nitrogen Dioxide and Mortality in 4 Countries
Source: JAMA Netw Open. 2024 Mar 1;7(3):e2354607. doi: 10.1001/jamanetworkopen.2023.54607 (PMC10907920; doi:10.1001/jamanetworkopen.2023.54607)
Supplement: Supplement 1. — eAppendix 1. Sources of Mortality and Population Data in Each Region eAppendix 2. Air Pollution Spatiotemporal Models for Jiangsu, China, Central-Southern Italy, and Germany eAppendix 3. Relative Humidity Calculation in Each Region eAppendix 4. Selection of the Number of Common Time-Varying Factors in the Unmeasured Time-Varying Spatial Unit Effect in the Interactive Fixed-Effects Model eAppendix 5. Sample R Code for the Main Interactive Fixed-Effects Model eAppendix 6. Classification of Urban and Rural Areas in Each Study Region eAppendix 7. Traditional 2-Stage Time-Series Analysis eFigure 1. Randomization Test for the Main Lag in Each Study Region eFigure 2. Plot of Estimated Common Time-Varying Factors and Unmeasured Time-Varying Spatial Unit Effects in the Main Single-Pollutant Models for PM2.5 or NO2 for Jiangsu, China, and California eFigure 3. Association Between Air Pollution and Mortality Estimated From Interactive Fixed-Effects Model and Traditional 2-Stage Time-Series Analysis eTable 1. Estimated Change in Daily Mortality Rate (per 100 000 People) Associated With a 10-µg/m3 Increase in PM2.5 or NO2 Concentration (95% CI) by Lag eTable 2. Estimated Change in Daily Mortality Rate (per 100 000 People) Associated With a 10-µg/m3 Increase in PM2.5 or NO2 Concentration by Urbanicity (95% CI) eTable 3. Estimated Changes in Daily Mortality Rate (per 100 000 People) Associated With a 10-µg/m3 Increase in PM2.5 and NO2 Concentrations in Sensitivity Analyses eReferences. [file jamanetwopen-e2354607-s001.pdf]

Supplementary Online Content

Ma Y, Nobile F, Marb A, et al. Short-term exposure to fine particulate matter and nitrogen dioxide and mortality in 4 countries. *JAMA Netw Open*. 2024;7(3):e2354607. doi:10.1001/jamanetworkopen.2023.54607

eAppendix 1. Sources of Mortality and Population Data in Each Region

eAppendix 2. Air Pollution Spatiotemporal Models for Jiangsu, China, Central-Southern Italy, and Germany

eAppendix 3. Relative Humidity Calculation in Each Region

eAppendix 4. Selection of the Number of Common Time-Varying Factors in the Unmeasured Time-Varying Spatial Unit Effect in the Interactive Fixed-Effects Model

eAppendix 5. Sample R Code for the Main Interactive Fixed-Effects Model

eAppendix 6. Classification of Urban and Rural Areas in Each Study Region

eAppendix 7. Traditional 2-Stage Time-Series Analysis

eFigure 1. Randomization Test for the Main Lag in Each Study Region

eFigure 2. Plot of Estimated Common Time-Varying Factors and Unmeasured Time-Varying Spatial Unit Effects in the Main Single-Pollutant Models for $PM_{2.5}$ or NO_2 for Jiangsu, China, and California

eFigure 3. Association Between Air Pollution and Mortality Estimated From Interactive Fixed-Effects Model and Traditional 2-Stage Time-Series Analysis

eTable 1. Estimated Change in Daily Mortality Rate (per 100 000 People) Associated With a 10-μg/m^3 Increase in $PM_{2.5}$ or NO_2 Concentration (95% CI) by Lag

eTable 2. Estimated Change in Daily Mortality Rate (per 100 000 People) Associated With a 10-μg/m^3 Increase in $PM_{2.5}$ or NO_2 Concentration by Urbanicity (95% CI)

eTable 3. Estimated Changes in Daily Mortality Rate (per 100 000 People) Associated With a 10-μg/m^3 Increase in $PM_{2.5}$ and NO_2 Concentrations in Sensitivity Analyses

eReferences.

This supplementary material has been provided by the authors to give readers additional information about their work.

© 2024 Ma Y et al. *JAMA Network Open*.

eAppendix 1. Sources of Mortality and Population Data in Each Region

Mortality data source in each region

Region	Source	Time period	Spatial resolution
Jiangsu, China	Jiangsu Provincial Center for Disease Prevention and Control	2015-2019	county
California, U.S.	California Comprehensive Death File, California Department of Public Health	2015-2019	county
Central-southern Italy	Italian National Institute of Statistics	2015-2019	municipality
Germany	Statistical Offices of the Federal States of Germany[a]	2015-2019	county

[a]In Germany, the mortality data are only accessible at the Research Data Center of the Statistical Offices of Germany

Population data source in each region

Region	Source	Time period	Spatial resolution
Jiangsu, China	China Statistical Yearbook	2015-2019	county
California, U.S.	American Community Survey 5-Year Data	2015-2019	county
Central-southern Italy	Italian National Institute of Statistics	2019[a]	municipality
Germany	German Federal Statistical Office	2015-2019	county

[a]The 2019 population data were used for all years in Central-southern Italy.

© 2024 Ma Y et al. *JAMA Network Open*.

eAppendix 2. Air Pollution Spatiotemporal Models for Jiangsu, China, Central-Southern Italy, and Germany

For Jiangsu, China, we obtained the spatiotemporal models at 1 × 1 km^2 resolution for NO_2 and $PM_{2.5}$, 2015-2019, that were developed by Dr. Meng Wang's team from University at Buffalo.[1,2] Briefly, the models incorporated variables from satellite, chemical transport model, land cover, elevation and slope, road network, urban density, and meteorological data to predict daily concentrations of air pollution in China, and utilized machine-learning algorithms, including random forests, extremely randomized trees, and extreme gradient boosting. Across the country, the overall cross-validation R^2 was 0.72 and 0.88 for NO_2 and $PM_{2.5}$, respectively.

Stafoggia et al.[3,4] estimated the daily $PM_{2.5}$ and NO_2 concentrations, in addition to concentrations of other pollutants, for 2015-2019 at 1 × 1 km^2 resolution using a random forest machine learning approach in Italy. For $PM_{2.5}$ only, a stage 1 was carried out to expand the number of stations and measuring points, based on the collocated PM_{10}. Thereafter, separately for each year and for each pollutant, a random forest model was trained with daily concentrations of the pollutant as the response variable and spatial and temporal variables such as climate zone, land cover, population, elevation, aerosol optical depth, daily mean air temperature, sea-level barometric pressure, precipitations, relative humidity, wind speed and direction, planetary boundary layer height, normalized difference vegetation index, and desert dust advection days, as predictors.

We obtained data for NO_2 and $PM_{2.5}$ in Germany from the German Environment Agency at a spatial resolution of 2 x 2 km^2 for 2015-2019. The data are based on a data assimilation technique known as Optimal Interpolation,[5] which uses the chemical REM-CALGRID model and integrates measured air pollutant concentrations from background monitoring stations. The final results are nationwide air quality maps based on information both of the observed and the modeled fields.[6] To adapt the data to the requirements of the INSPIRE Directive (a European Union spatial data infrastructure) and to make it more comparable to the other regions in this project, the data were downscaled to a resolution of 1x1 km^2 by bilinear interpolation.

© 2024 Ma Y et al. *JAMA Network Open*.

eAppendix 3. Relative Humidity Calculation in Each Region

For Jiangsu, China; California, U.S.; and Central-southern Italy, dew point temperature data at 0.1° × 0.1° resolution were extracted from the ERA5-Land reanalysis dataset[7] and were averaged to daily spatial-unit level. In these three regions, daily relative humidity (RH) was calculated based on the information on air temperature and dew point temperature by the following steps.

Step 1. Calculate the vapor pressure (e)

The vapor pressure e was calculated from a given dew point T_d (in K) by using the Clausius-Clapeyron relation:

$$e(T_d) = e_s(T_0) \times \exp(\frac{L}{R_w}(\frac{1}{T_0} - \frac{1}{T_d}))$$

where $e_s(T_0) = 6.11\text{hPa}$ is the saturation vapor pressure at a reference temperature $T_0 = 273.15\text{K}$; $L = 2.5 \times 106\, J/kg$ is the latent heat of evaporation for water; and $R_w = \frac{1000R}{M_w} = 461.52\, J/(kgK)$ is the specific gas constant for water vapor (where $R = 8.3144621\, J/(molK)$ is the molar gas constant and $M_w = 18.01528\ g/mol$ is the molar mass of water vapor). For more details refer to Shaman and Kohn (2009).[8]

Step 2. Calculate the saturation vapor pressure (e_s)

The saturation vapor pressure e_s was calculated from a given air temperature T (in K) by the same equation:

$$e(T) = e_s(T_0) \times \exp(\frac{L}{R_w}(\frac{1}{T_0} - \frac{1}{T}))$$

Step 3. Calculate the relative humidity (RH)

Finally, we calculated the relative humidity RH using the vapor pressure and saturation vapor pressure values:

$$RH = \frac{e}{e_s} \times 100$$

The calculation of RH in these three regions was performed with R package *humidity*.[9]

For Germany, daily mean RH at a spatial resolution of 1 x 1 km^2 was derived from spatiotemporal models for the years 2015-2019. In brief, the German-wide RH was predicted by applying a random forest model which incorporated data from multiple sources such as ground-based RH observations, modeled air temperature, wind speed, and precipitation as well as remote sensing elevation, greenness, and the visible light bands (red, green and blue).[10] The date information was also added to represent the temporal variability of the relationship between the response and predictor variables. The 10-fold cross-validation results of the random forest model showed high performance (R^2 = 0.80 and Root Mean Square Error (RMSE) of 5.42 %) and they were externally confirmed by a comparison against an independent and dense monitoring network in the Augsburg region ($R^2 \geq 0.84$, RMSE ≤ 5.91 %).

© 2024 Ma Y et al. *JAMA Network Open*.

eAppendix 4. Selection of the Number of Common Time-Varying Factors in the Unmeasured Time-Varying Spatial Unit Effect in the Interactive Fixed-Effects Model

The number of common time-varying factors $f_{l,t}$ in the interactive fixed effects model was selected based on the criteria proposed by Bai and Ng (2002).[11] The number of factors $\hat{d}$ can be obtained by minimizing the following criterion:

$$PC(l) = \frac{1}{nT}\sum_{i=1}^{n}\sum_{t=1}^{T}(y_{i,t} - \hat{y}_{i,t}(l))^2 + lg_{n,T}$$

for all $l \in \{1,2,\dots\}$, where n is the number of spatial units, T is the number of days, and $\hat{y}_{i,t}(l)$ is the fitted value for a given factor dimension l. $g_{n,T}$ is a penalty term, which penalizes the undesired variance reduction caused by an increasing number of factors $\hat{d}$. This penalty term can be estimated by

$$g_{n,T} = \hat{\sigma}^2 \frac{(n+T)}{nT} \log\left(\frac{nT}{n+T}\right)$$

where $\hat{\sigma}^2$ is the sample variance estimator of the residuals $\varepsilon_{i,t}$. This variance estimator $\hat{\sigma}^2$ can be obtained by

$$\hat{\sigma}^2(d_{max}) = \frac{1}{nT}\sum_{i=1}^{n}\sum_{t=1}^{T}(y_{i,t} - \hat{y}_{i,t}(d_{max}))^2$$

where d_{max} is an arbitrary maximal dimension of factors that is greater than d. In our study, we set d_{max} as the square root of the number of spatial units in each study region. All statistical analyses were conducted with R software (version 4.1.3) using the package *phtt.*[12]

© 2024 Ma Y et al. *JAMA Network Open*.

eAppendix 5. Sample R Code for the Main Interactive Fixed-Effects Model

```
## load packages
library(phtt)
library(splines)

## data preparation
n.date <- length(unique(data$time)) # number of total days -1
n.county <- length(unique(data$countycode)) # number of spatial units in each study region

Total.rate <- matrix(data$Total.rate.dif, n.date, n.county) # mortality rate after first-order differencing
NO2 <- matrix(data$NO2.lag.dif, n.date, n.county)
PM25 <- matrix(data$PM25.lag.dif, n.date, n.county)

Tmean.spline <- ns(data$TMP.lag.dif, df=5)
Tmean.ns1 <- matrix(Tmean.spline[,1], n.date, n.county)
Tmean.ns2 <- matrix(Tmean.spline[,2], n.date, n.county)
Tmean.ns3 <- matrix(Tmean.spline[,3], n.date, n.county)
Tmean.ns4 <- matrix(Tmean.spline[,4], n.date, n.county)
Tmean.ns5 <- matrix(Tmean.spline[,5], n.date, n.county)

## main model for NO2
model.NO2 <- Eup(Total.rate ~ NO2 + Tmean.ns1 + Tmean.ns2 + Tmean.ns3 + Tmean.ns4 +
Tmean.ns5, additive.effects = "individual")

## main model for PM2.5
model.PM25 <- Eup(Total.rate ~ PM25 + Tmean.ns1 + Tmean.ns2 + Tmean.ns3 + Tmean.ns4 +
Tmean.ns5, additive.effects = "individual")
```

© 2024 Ma Y et al. *JAMA Network Open*.

eAppendix 6. Classification of Urban and Rural Areas in Each Study Region

Region	Classification standard	# of urban and rural spatial units	Note
Jiangsu, China	Chinese National Population Census 2010	Urban: 41<br>Rural: 41	Each county was categorized as “Urban” or “Rural” based on the median of the percentage of urban residents across Jiangsu counties.[13]
California, U.S.	U.S. National Center for Health Statistics Urban-Rural Classification Scheme for Counties	Urban: 14<br>Rural: 18	The categories “Large central metro” and “Large fringe metro” were combined into one single “Urban” category. The other four categories (Medium metro, Small metro, Micropolitan, and Non-core) were classified as "Rural."
Central-southern Italy	Eurostat Degree of Urbanization	Urban: 104<br>Rural: 565	The category labeled as "Cities" was classified as “Urban.” The categories "Towns and suburbs" and "Rural areas" were combined to form a single "Rural" category.
Germany	German Federal Institute for Research on Building, Urban Affairs and Spatial Development Settlement Structural County Types	Urban: 199<br>Rural: 202	“Independent cities” and “Urban counties” were combined to a single "Urban" category; “Rural counties with agglomeration” and “Rural counties” were combined to the "Rural" category.

© 2024 Ma Y et al. *JAMA Network Open*.

eAppendix 7. Traditional 2-Stage Time-Series Analysis

We compared the results from the IFE model with those from a traditional two-stage time-series model, with model settings similar to those in previous studies.[14,15]

In the first stage, we estimated the association of $PM_{2.5}$ or NO_2 with mortality for each county or municipality using a quasi-Poisson generalized linear model, controlling for seasonality and long-term trend, day of the week, and air temperature:

$$\log[\mathrm{E}(\text{Mortality count})] = \mu + \beta_1 \text{Air pollution} + \beta_2 \text{DOW} + \text{ns}(\text{date}, \text{df} = 6 \times \text{years}) + \text{ns}(\text{Temperature}, \text{df} = 5)$$

where $\mathrm{E}(\text{Mortality count})$ is the daily morality count in each spatial unit; μ is the intercept; Air pollution is the daily $PM_{2.5}$ or NO_2 concentration in each spatial unit; DOW is an indicator of the day of the week; the seasonality and long-term trend were controlled by a natural cubic spline over the range of study dates with 6 degrees of freedom (df) per year; and air temperature on the same lag as air pollution was controlled by a natural cubic spline with 5 dfs.

In the second stage, a univariate random-effect meta-analysis was used to pool the county/ municipality-specific risk estimates into a single summary estimate of association in each study region. As in the main IFE model, we explored the lag pattern on the current day and the previous two days using both single lag days (lag0 to lag2) and cumulative lag days (lag01 to lag02), and the main lag was defined as the lag with the greatest effect size.

© 2024 Ma Y et al. *JAMA Network Open*.

eFigure 1. Randomization Test for the Main Lag in Each Study Region

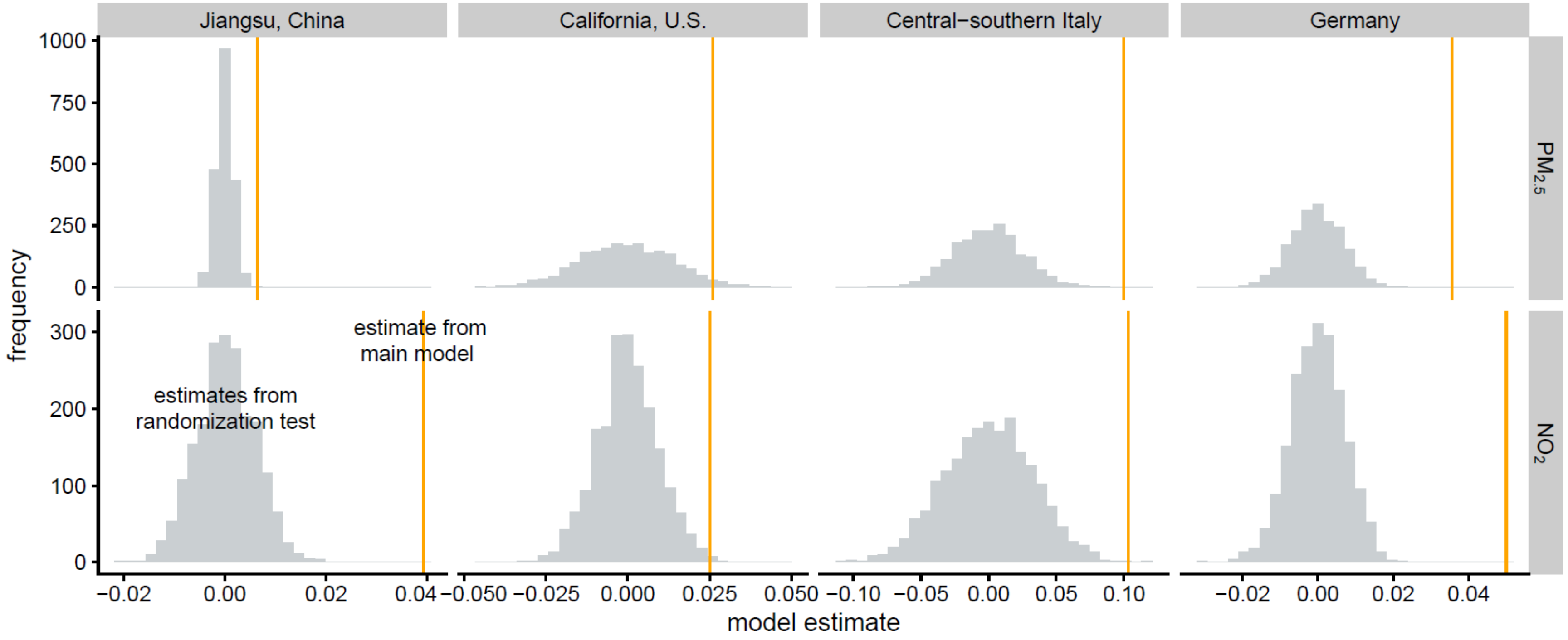

This figure shows the distribution of the model estimates for main lags when the $PM_{2.5}$ or NO_2 concentration was randomized 2,000 times across days in each spatial unit. The distributions of the estimates from the randomization tests (the histograms) were approximately centered at zero and the coefficient estimates from our single-pollutant models (the orange lines) for the main lag for all study regions fell substantially outside these distributions, indicating that the estimated associations between changes in air pollution and changes in mortality rate in these regions were unlikely driven by temporal dependence due to a model misspecification.

© 2024 Ma Y et al. *JAMA Network Open*.

eFigure 2. Plot of Estimated Common Time-Varying Factors and Unmeasured Time-Varying Spatial Unit Effects in the Main Single-Pollutant Models for $PM_{2.5}$ or NO_2 for Jiangsu, China, and California

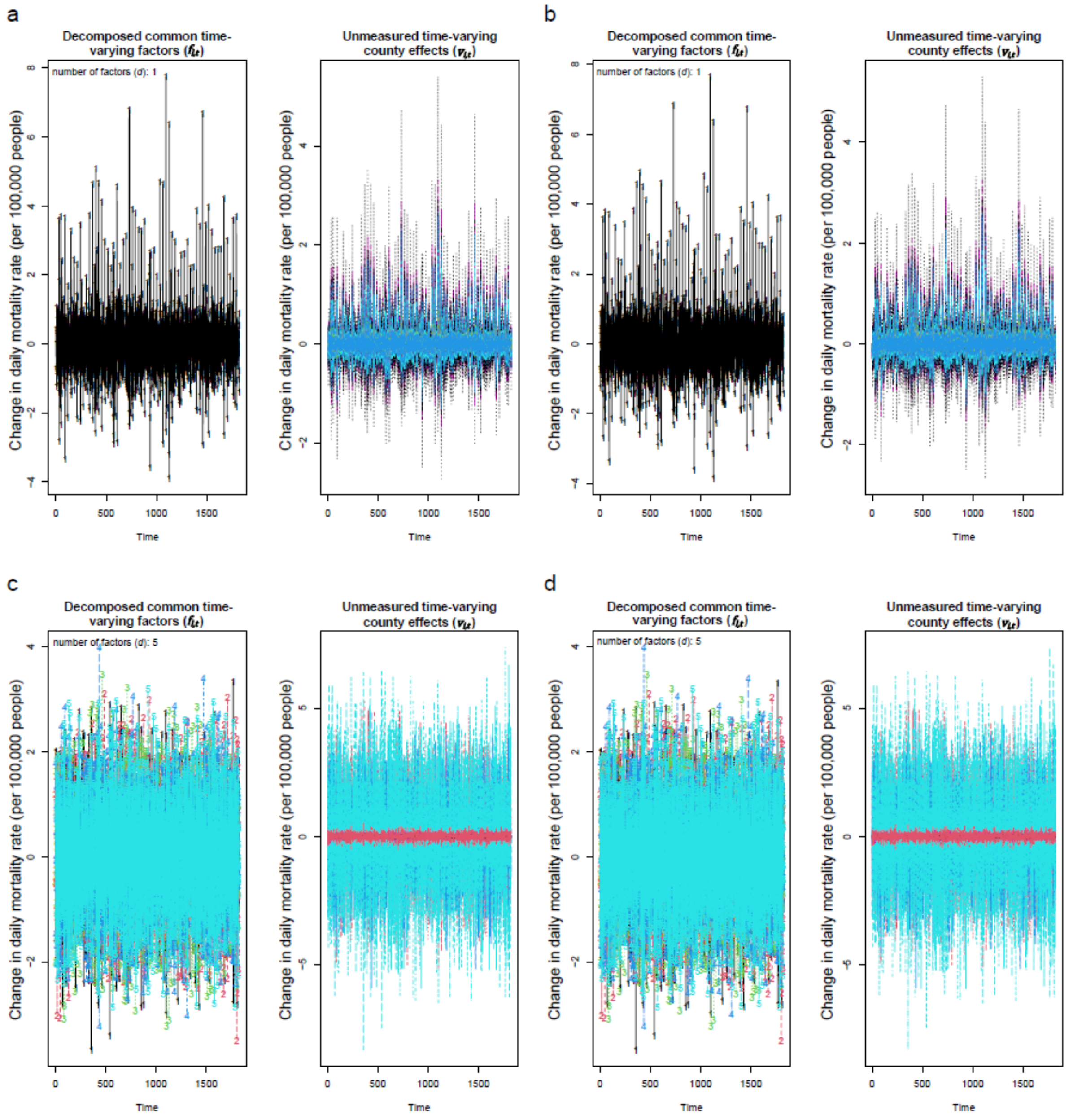

This plot visualizes the estimated common time-varying factors ($f_{l,t}$) and the unmeasured time-varying county effects ($v_{i,t}$) in Jiangsu, China (a: $PM_{2.5}$ lag01; b: NO_2 lag02) and California, U.S. (c: $PM_{2.5}$ lag02; d: NO_2 lag02). $v_{i,t}$ is decomposed into d common time-varying factors $f_{l,t}$, with corresponding unobserved spatial unit level loading parameters $\lambda_{i,l}$ ($v_{i,t} = \sum_{l=1}^{d} \lambda_{i,l} f_{l,t}$). d represents the estimated number of common time-varying factors in the unmeasured time-varying county effect (this spatial unit effect was estimated to be 0 in Central-southern Italy and Germany). d was selected following the criteria proposed by Bai and Ng (2002) (see details in eAppendix 4). In the plots of the decomposed common time-varying factors, each color represents each decomposed common time-varying factor; in the plots of the unmeasured time-varying county effects, each color represents each county.

© 2024 Ma Y et al. *JAMA Network Open*.

eFigure 3. Association Between Air Pollution and Mortality Estimated From Interactive Fixed-Effects Model and Traditional 2-Stage Time-Series Analysis

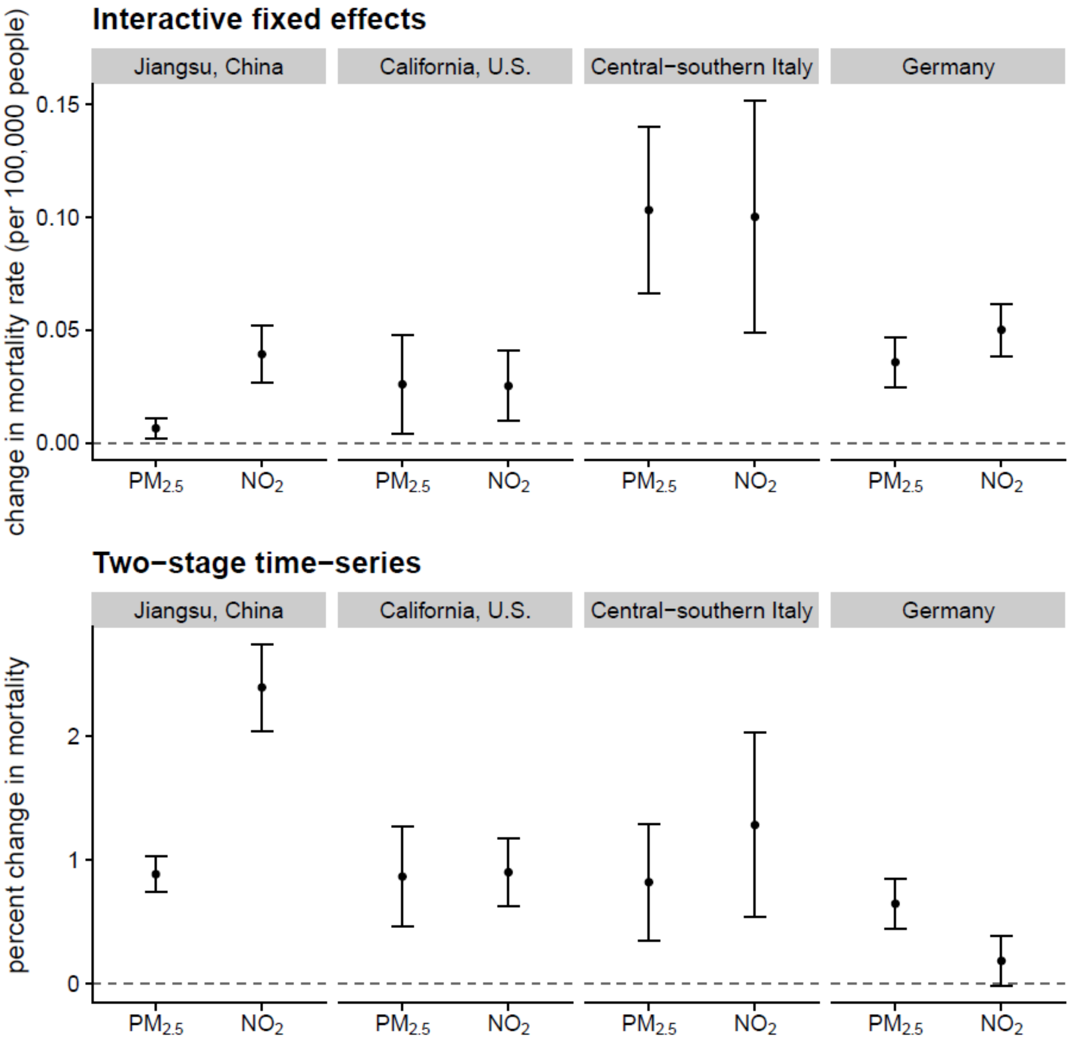

This plot compares the results from the main interactive fixed effects model and the comparative traditional two-stage time-series analysis on the main lag (the lag with the greatest effect size). The estimates from the two models have different interpretations: the estimates from the IFE model represent the estimated change in daily mortality rate (per 100,000 people) associated with a 10 µg/m^3 increase in $PM_{2.5}$ or NO_2 concentrations, while the estimates from the time-series analysis represent the percent change in mortality for the same increase in $PM_{2.5}$ or NO_2. The results from both models suggested that short-term exposure to $PM_{2.5}$ and NO_2 was associated with an increase in all-cause mortality.

Main lag in the IFE model: Jiangsu, China: $PM_{2.5}$ lag01, NO_2 lag02; California, U.S.: $PM_{2.5}$ lag02, NO_2 lag02; Central-southern Italy: $PM_{2.5}$ lag02, NO_2 lag02; Germany: $PM_{2.5}$ lag01, NO_2 lag01

Main lag in the time-series model: Jiangsu, China: $PM_{2.5}$ lag02, NO_2 lag02; California, U.S.: $PM_{2.5}$ lag02, NO_2 lag2; Central-southern Italy: $PM_{2.5}$ lag2, NO_2 lag2; Germany: $PM_{2.5}$ lag02, NO_2 lag1

© 2024 Ma Y et al. *JAMA Network Open*.

eTable 1. Estimated Change in Daily Mortality Rate (per 100 000 People) Associated With a 10-μg/m^3 Increase in $PM_{2.5}$ or NO_2 Concentration (95% CI) by Lag

Lag	Single-pollutant model		Two-pollutant model	
	$PM_{2.5}$	NO_2	$PM_{2.5}$	NO_2
Jiangsu, China				
Lag0	0.00 (0.00, 0.01)	0.01 (0.00, 0.02)	0.00 (0.00, 0.00)	0.01 (0.00, 0.02)
Lag1	0.00 (0.00, 0.01)	0.02 (0.01, 0.03)	0.00 (-0.01, 0.00)	0.02 (0.01, 0.03)
Lag2	0.00 (-0.01, 0.00)	0.00 (-0.01, 0.01)	0.00 (-0.01, 0.00)	0.00 (-0.01, 0.01)
Lag01	0.01 (0.00, 0.01)	0.04 (0.02, 0.05)	0.00 (-0.01, 0.00)	0.04 (0.03, 0.05)
Lag02	0.01 (0.00, 0.01)	0.04 (0.03, 0.05)	-0.01 (-0.01, 0.00)	0.05 (0.03, 0.07)
California, U.S.				
Lag0	0.01 (0.00, 0.03)	0.01 (-0.01, 0.02)	0.01 (-0.01, 0.02)	0.00 (-0.01, 0.01)
Lag1	0.02 (0.00, 0.03)	0.01 (0.00, 0.02)	0.01 (0.00, 0.03)	0.01 (-0.01, 0.02)
Lag2	-0.01 (-0.02, 0.01)	0.00 (-0.01, 0.01)	-0.01 (-0.02, 0.01)	0.01 (-0.01, 0.02)
Lag01	0.02 (0.00, 0.04)	0.01 (0.00, 0.02)	0.02 (0.00, 0.04)	0.01 (-0.01, 0.02)
Lag02	0.03 (0.00, 0.05)	0.03 (0.01, 0.04)	0.02 (0.00, 0.04)	0.02 (0.00, 0.04)
Central-southern Italy				
Lag0	0.03 (0.00, 0.05)	0.03 (-0.01, 0.06)	0.02 (-0.01, 0.05)	0.01 (-0.03, 0.05)
Lag1	0.05 (0.03, 0.08)	0.00 (-0.03, 0.04)	0.07 (0.03, 0.10)	-0.04 (-0.08, 0.00)
Lag2	0.01 (-0.02, 0.03)	0.04 (0.00, 0.07)	-0.01 (-0.04, 0.02)	0.04 (0.00, 0.08)
Lag01	0.08 (0.05, 0.11)	0.04 (-0.01, 0.08)	0.09 (0.05, 0.13)	-0.03 (-0.08, 0.02)
Lag02	0.10 (0.07, 0.14)	0.10 (0.05, 0.15)	0.09 (0.05, 0.14)	0.03 (-0.04, 0.09)
Germany				
Lag0	0.01 (0.00, 0.02)	0.03 (0.02, 0.04)	0.00 (-0.01, 0.01)	0.03 (0.02, 0.04)
Lag1	0.02 (0.01, 0.03)	0.01 (0.00, 0.02)	0.02 (0.01, 0.03)	0.01 (-0.01, 0.02)
Lag2	-0.02 (-0.03, -0.01)	-0.02 (-0.03, -0.01)	-0.02 (-0.03, -0.01)	-0.01 (-0.02, 0.00)
Lag01	0.04 (0.02, 0.05)	0.05 (0.04, 0.06)	0.02 (0.01, 0.03)	0.04 (0.03, 0.05)
Lag02	0.02 (0.00, 0.03)	0.04 (0.03, 0.06)	0.00 (-0.01, 0.02)	0.04 (0.03, 0.06)

© 2024 Ma Y et al. *JAMA Network Open*.

eTable 2. Estimated Change in Daily Mortality Rate (per 100 000 People) Associated With a 10-µg/m^3 Increase in $PM_{2.5}$ or NO_2 Concentration by Urbanicity (95% CI)[a]

Urbanicity	Single-pollutant model				Two-pollutant model			
	$PM_{2.5}$	*P* value[b]	NO_2	*P* value	$PM_{2.5}$	*P* value	NO_2	*P* value
Jiangsu, China								
Urban	0.01 (0.00, 0.01)	.76	0.03 (0.02, 0.04)	.07	0.00 (-0.01, 0.00)	.22	0.04 (0.02, 0.05)	.02
Rural	0.01 (0.00, 0.01)		0.05 (0.03, 0.07)		-0.01 (-0.01, 0.00)		0.07 (0.04, 0.10)	
California, U.S.								
Urban	0.02 (0.00, 0.04)	.74	0.01 (0.00, 0.02)	.16	0.02 (-0.01, 0.04)	.96	0.01 (-0.01, 0.02)	.31
Rural	0.03 (-0.01, 0.06)		0.04 (0.00, 0.08)		0.02 (-0.02, 0.05)		0.03 (-0.01, 0.07)	
Central-southern Italy								
Urban	0.07 (0.01, 0.13)	.22	0.09 (0.02, 0.15)	.79	0.04 (-0.04, 0.11)	.10	0.06 (-0.02, 0.15)	.36
Rural	0.12 (0.07, 0.16)		0.10 (0.03, 0.18)		0.11 (0.06, 0.17)		0.01 (-0.09, 0.10)	
Germany								
Urban	0.03 (0.02, 0.05)	.83	0.05 (0.03, 0.06)	.36	0.02 (0.00, 0.03)	.55	0.04 (0.03, 0.05)	.72
Rural	0.04 (0.02, 0.06)		0.06 (0.03, 0.09)		0.02 (0.00, 0.04)		0.05 (0.01, 0.08)	

[a]In stratified analyses, we used the lag day with the greatest pollutant-mortality association for each pollutant in each region (Jiangsu, China: $PM_{2.5}$ lag01, NO_2 lag02; California, U.S.: $PM_{2.5}$ lag02, NO_2 lag02; Central-southern Italy: $PM_{2.5}$ lag02, NO_2 lag02; Germany: $PM_{2.5}$ lag01, NO_2 lag01).

[b]We tested the statistical differences in effect estimates between urban and rural areas by calculating *P* values based on the z score derived from the coefficients and standard errors for urban vs. rural.

© 2024 Ma Y et al. *JAMA Network Open*.

eTable 3. Estimated Changes in Daily Mortality Rate (per 100 000 People) Associated With a 10-µg/m^3 Increase in $PM_{2.5}$ and NO_2 Concentrations in Sensitivity Analyses[a]

Air pollutant	Jiangsu, China	California, U.S.	Central-southern Italy	Germany
$PM_{2.5}$				
Main model	0.01 (0.00, 0.01)	0.03 (0.00, 0.05)	0.10 (0.07, 0.14)	0.04 (0.02, 0.05)
Adjust for RH	0.01 (0.00, 0.01)	0.03 (0.00, 0.05)	0.11 (0.07, 0.14)	0.03 (0.02, 0.04)
Adjust for dew point	0.01 (0.00, 0.01)	0.03 (0.00, 0.05)	0.11 (0.07, 0.14)	0.03 (0.02, 0.05)
Traditional fixed effects	0.01 (0.00, 0.01)	0.02 (0.00, 0.05)	0.10 (0.07, 0.14)	0.04 (0.02, 0.05)
Temperature df=4	0.01 (0.00, 0.01)	0.02 (0.00, 0.05)	0.10 (0.07, 0.14)	0.04 (0.02, 0.05)
Temperature df=6	0.01 (0.00, 0.01)	0.03 (0.00, 0.05)	0.10 (0.07, 0.14)	0.04 (0.02, 0.05)
NO_2				
Main model	0.04 (0.03, 0.05)	0.03 (0.01, 0.04)	0.10 (0.05, 0.15)	0.05 (0.04, 0.06)
Adjust for RH	0.03 (0.02, 0.05)	0.03 (0.01, 0.04)	0.10 (0.05, 0.15)	0.05 (0.04, 0.06)
Adjust for dew point	0.04 (0.02, 0.05)	0.02 (0.01, 0.04)	0.10 (0.05, 0.15)	0.05 (0.04, 0.06)
Traditional fixed effects	0.04 (0.03, 0.05)	0.03 (0.01, 0.04)	0.10 (0.05, 0.15)	0.05 (0.04, 0.06)
Temperature df=4	0.04 (0.03, 0.05)	0.03 (0.01, 0.04)	0.10 (0.05, 0.15)	0.05 (0.04, 0.06)
Temperature df=6	0.04 (0.03, 0.05)	0.02 (0.01, 0.04)	0.10 (0.05, 0.15)	0.05 (0.04, 0.06)

RH: relative humidity; df: degree of freedom

[a]In sensitivity analyses, we used the lag day with the greatest pollutant-mortality association for each pollutant in each region (Jiangsu, China: $PM_{2.5}$ lag01, NO_2 lag02; California, U.S.: $PM_{2.5}$ lag02, NO_2 lag02; Central-southern Italy: $PM_{2.5}$ lag02, NO_2 lag02; Germany: $PM_{2.5}$ lag01, NO_2 lag01).

© 2024 Ma Y et al. *JAMA Network Open*.

eReferences.

1. Huang C, Hu J, Xue T, Xu H, Wang M. High-resolution spatiotemporal modeling for ambient PM2. 5 exposure assessment in China from 2013 to 2019. *Environ Sci Technol.* 2021;55(3):2152-2162.
2. Huang C, Sun K, Hu J, Xue T, Xu H, Wang M. Estimating 2013–2019 NO2 exposure with high spatiotemporal resolution in China using an ensemble model. *Environ Pollut.* 2022;292:118285.
3. Stafoggia M, Cattani G, Ancona C, Ranzi A. La valutazione dell’esposizione della popolazione italiana all’inquinamento atmosferico nel periodo 2016-2019 per lo studio della relazione tra inquinamento atmosferico e COVID-19 [Exposure assessment of air pollution in Italy 2016-2019 for future studies on air pollution and COVID-19]. *Epidemiol Prev.* 2020;44(5-6 Suppl 2):161-168.
4. Stafoggia M, Bellander T, Bucci S, et al. Estimation of daily PM(10) and PM(2.5) concentrations in Italy, 2013-2015, using a spatiotemporal land-use random-forest model. *Environ Int.* 2019;124:170-179.
5. Felmming J, Stern, R. *Datenassimilation auf der Basis der Optimalen Interpolation für die Kartierung von Immissionsbelastungen - Beschreibung der Methodik und praktische Anwendung für 2002 [Data assimilation on the basis of optimal interpolation for the mapping of immission loads - Description of the methodology and practical application for 2002].* Institut für Meteorologie, Troposphärische Umweltforschung, Freie Universität Berlin;2004.
6. German Environment Agency. Kartographische Darstellung der flächenhaften Immissionsbelastung in Deutschland durch Kombination von Messung und Rechnung [Cartographic representation of the areal immission load in Germany by combination of measurement and calculation]. 2018; https://www.umweltbundesamt.de/publikationen/kartografische-darstellung-flaechenhaften
7. Muñoz Sabater J. ERA5-Land hourly data from 1950 to present. In: Copernicus Climate Change Service (C3S) Climate Data Store (CDS), ed2019.
8. Shaman J, Kohn M. Absolute humidity modulates influenza survival, transmission, and seasonality. *Proc Natl Acad Sci U S A.* 2009;106(9):3243-3248.
9. Cai J. humidity: Calculate Water Vapor Measures from Temperature and Dew Point. *R package version 0.1.5.* 2019.
10. Nikolaou N, Bouwer L, Dallavalle M, et al. Spatiotemporally resolved daily relative humidity predictions across Germany during 2000-2021: a Random Forest approach. Paper presented at: ISEE Conference Abstracts2022.
11. Bai J, Ng S. Determining the number of factors in approximate factor models. *Econometrica.* 2002;70(1):191-221.
12. Bada O, Liebl D. phtt: panel data analysis with heterogeneous time trends in R. *J Stat Softw.* 2014;59(6):1-33.
13. Lin C, Ma Y, Liu R, et al. Associations between short-term ambient ozone exposure and cause-specific mortality in rural and urban areas of Jiangsu, China. *Environ Res.* 2022;211:113098.
14. Liu C, Chen R, Sera F, et al. Ambient particulate air pollution and daily mortality in 652 cities. *N Engl J Med.* 2019;381(8):705-715.
15. Meng X, Liu C, Chen R, et al. Short term associations of ambient nitrogen dioxide with daily total, cardiovascular, and respiratory mortality: multilocation analysis in 398 cities. *BMJ.* 2021;372:n534.

© 2024 Ma Y et al. *JAMA Network Open*.
