# Supplementary material for: Short-Term Exposure to Fine Particulate Matter and Nitrogen Dioxide and Mortality in 4 Countries
Source: JAMA Netw Open. 2024 Mar 1;7(3):e2354607. doi: 10.1001/jamanetworkopen.2023.54607 (PMC10907920; doi:10.1001/jamanetworkopen.2023.54607)
Supplement: Supplement 2. — Data Sharing Statement [file jamanetwopen-e2354607-s002.pdf]

Data Sharing Statement

Ma. Short-Term Exposure to Fine Particulate Matter and Nitrogen Dioxide and Mortality in 4 Countries. *JAMA Netw Open*. Published March 01, 2024.
doi:10.1001/jamanetworkopen.2023.54607

Data
Data available: No

Additional Information
Explanation for why data not available: The station-based air pollution data in California, U.S. are publicly available on the U.S. EPA website (https://www.epa.gov/outdoor-air-quality-data); the modelled air pollution data in Jiangsu, China, Central-southern Italy, and Germany are available upon request. The mortality data in Italy are publicly available on the website of Italian National Institute of Statistics (https://www.istat.it/en/); the mortality data in the other three regions are confidential.
